# Health economic evaluation of a web-based intervention for depression: the EVIDENT-trial, a randomized controlled study

**DOI:** 10.1186/s13561-019-0233-y

**Published:** 2019-06-07

**Authors:** Viola Gräfe, Thomas Berger, Martin Hautzinger, Fritz Hohagen, Wolfgang Lutz, Björn Meyer, Steffen Moritz, Matthias Rose, Johanna Schröder, Christina Späth, Jan Philipp Klein, Wolfgang Greiner

**Affiliations:** 1 Department of Health Economics and Health Care Management, School of Public Health, Bielefeld University, Universitätsstraße 25, 33615 Bielefeld, Germany; 2 Department of Clinical Psychology and Psychotherapy, University of Bern, Hochschulstrasse 6, 3012 Bern, Switzerland; 3 Department of Psychology, Eberhard Karls University Tuebingen, Schleichstraße 4, 72076 Tuebingen, Germany; 4 Department of Psychiatry and Psychotherapy, University of Luebeck, Ratzeburger Allee 160, 23538 Luebeck, Germany; 5 Department of Psychology, University of Trier, Am Wissenschaftspark 25, +2754296 Trier, Germany; 6 Research Department, Gaia AG, Hans-Henny-Jahnn-Weg, 5322085 Hamburg, Germany; 7 The Department of Psychiatry and Psychotherapy, University Medical Center Hamburg-Eppendorf, Martinistraße 52, 20246 Hamburg, Germany; 8 Department of Psychosomatic Medicine, Charité University Medical Center, Hindenburgdamm 30, 12200 Berlin, Germany; 9 The Institute for Sex Research and Forensic Psychiatry, University Medical Center Hamburg-Eppendorf, Martinistraße 52, 20246 Hamburg, Germany

**Keywords:** Economic issues, Outcome studies, Health economic evaluation, E-mental-health, Deprexis, Depression, Randomized controlled trial

## Abstract

**Background:**

Depression often remains undiagnosed or treated inadequately. Web-based interventions for depression may improve accessibility of treatment and reduce disease-related costs. This study aimed to examine the potential of the web-based cognitive behavioral intervention “deprexis” in reducing disease-related costs.

**Methods:**

Participants with mild to moderate depressive symptoms were recruited and randomized to either a 12-week web-based intervention (deprexis) in addition to care as usual (intervention group) or care as usual (control group). Outcome measures were health-related resource use, use of medication and incapacity to work as well as relating direct health care costs. Outcomes were assessed on patients’ self-report at baseline, three months and six months.

**Results:**

A total of 1013 participants were randomized. In both groups total direct health care costs decreased during the study period, but changes from baseline did not significantly differ between study groups. Numeric differences between study groups existed in outpatient treatment costs. They could be attributed to differences in changes of costs for psychotherapeutic treatment from baseline. Whereas costs for psychotherapeutic treatment decreased in the intervention group, costs increased in the control group (− 16.8% (€80) vs. + 14.7% (€60)) (t_df = 685_ = 2.57; *p* = 0.008).

**Conclusion:**

The study indicates the health economic potential of innovative e-mental-health programs. There is evidence to suggest that the use of deprexis over a period of 12 weeks leads to a decrease in outpatient treatment cost, especially in those related to different types of psychotherapeutic treatment.

## Background

Major depression is a worldwide health problem, which lowers quality of life for the individual and generates huge costs for society. The lifetime prevalence of a diagnosed depression is estimated at 11.6% to 13.0% in German adults, with women having a nearly twice as high risk of disease as compared to men [1–5]. From a societal perspective, depressive disorders are associated with a substantial loss of resources. The diagnosis of depression has become the second most important reason for an incapacity for work [6–8]. In comparison to people without depression, patients with depressive disorder report twice as many days of incapacity for work [9]. Therefore, employees had an average absence of 51.8 days due to depressive episodes in 2014 [10]. In addition to indirect costs due to disease related productivity losses, depressive disorders are associated with high health care costs. Thus, the estimated annual direct treatment costs for Germany range between €686 and €3849 per patient within different studies. The total direct costs of depression in Germany were estimated at 5,2 billion Euro for the whole population [5, 11].

A depressive episode needs to be treated if symptoms exceed a certain period, persistence and strength [12]. But despite differentiated guidelines and a well-developed health care system, depressive episodes are rarely identified in time and treated adequately. Thus, many individuals with depression remain untreated, even in countries with well-developed health care systems [13]. This globally documented treatment gap in the management of mental illnesses may be counteracted by internet based self-help interventions. This form of intervention is particularly relevant as a treatment of mild to moderate depression [14, 15]. Employed in a stepped-care model, low intensity online-based interventions may bridge the treatment gap as an appropriate first option for patients with mild to moderate depressive symptoms. Advantages are low threshold, local and temporal independence, reductions in waiting time for face-to-face treatment, empowerment and anonymity [15, 16].

During the recent years different studies along randomized controlled trials as well as some meta-analyses provided evidence for the clinical effectiveness of e-mental health interventions (especially in the treatment of mild to moderate depressive symptoms). For example, a meta-analysis by Cujipers and colleagues stated that that self-guided psychological treatment has a small but statistically significant effect on participants with elevated levels of depressive symptomatology [14]. Moreover, another meta-analysis that was conducted in this textual context underlined the effectiveness of web-based interventions in the treatment of depression.

The meta-analysis by Karyotaki et al. demonstrated self-guided internet-based behavioral therapy to be significantly more effective on depressive symptoms severity and treatment response in comparison to control conditions [17].

While there is strong evidence for the effectiveness of web-based treatments for depression, effects on overall health care costs have been less well researched. In this context, only a few health economic evaluations exist up to now, most of them evaluating guided, less commonly unguided or minimally-guided internet interventions. Whereas most studies indicated that guided web-based interventions have the potential to be cost-effective [18], health economic evaluations of self-guided treatment programs tend to classify those interventions as not cost-effective according to direct costs of health services or productivity losses [19–21].

Against this background, the present study was designed to examine, whether the use of the minimally-guided cognitive behavioral self-help program deprexis over a period of 12 weeks in addition to care as usual leads to a significant reduction in direct health care costs within six months of observation. The main results of this study, the EVIDENT-trial, has been published elsewhere [22].

## Methods

### Study design

The EVIDENT-Trial is as a prospective, parallel-group, multicenter, randomized, controlled and assessor-blinded study which was conducted between August 2013 and December 2014. The study was approved by the Ethics Committee of the German Psychological Association (reference-number SM 04_2012) and registered at ClinicalTrials.gov (identifier: NCT01636752). A study protocol with a detailed description of the trial design has been published [23].

Using an a-priori generated allocation schedule with random numbers, all participants were randomized into either an intervention group or a control group. Participants of the intervention group gained access to the online based self-help program “deprexis” for a period of 12 weeks in addition to care as usual. The program aims to promote self-management skills as well as to empower people with depressive symptoms to learn new and healthier behaviors. It consists of ten different modules, covering a broad range of elements of cognitive behavioral therapy such as behavioral activation and cognitive modification, psychoeducation, mindfulness and acceptance or interpersonal skills. For a detailed description of the program and all of its modules see Meyer et al. [24].

The control group received care as usual and was permitted to use any kind of therapy or treatment offered in standard care under the statutory health insurance scheme (e.g. outpatient medical care, inpatient hospital care or pharmaceutical care as well as psychiatric or psychotherapeutic treatment). After taking part in the last follow-up assessment, the participants of the control group were also invited to use the internet based program for a 12-week period.

### Participants

Participants were recruited from various settings, including inpatient and outpatient medical and psychological clinics, health insurance companies, online forums for depression as well as different media (e. g. newspaper and radio) between August 2012 and December 2013.

Inclusion criteria were the presence of mild to moderate depressive symptoms, defined by scores between 5 and 14 on the Patient Health Questionnaire-9 (PHQ-9), age between 18 and 65 years, an adequate command of the German language, the availability of internet access and electronically written informed consent, which was obtained online prior to baseline assessment.

People with moderately severe to severe depressive symptoms (PHQ-9 score > 14), an acute suicidal tendency (> 0, PHQ-9 Item 9), a diagnosis of bipolar disorder or lifetime schizophrenia (both determined by a diagnostic telephone interview) or other serious mental or physical illnesses that required acute treatment were excluded from the study.

### Resource use and costing

The health economic evaluation of the EVIDENT-trial focused on healthcare utilization, medication use and incapacity for work due to illness, as well as on resulting direct costs. Estimates of direct costs were derived from the payer-perspective. Therefore, patients’ time costs, traveling costs as well as indirect costs due to absenteeism or presentism were not included in the analysis. Fees for the use of the web based program were excluded from this analysis, as these are negotiated individually with clients such as health insurance companies and vary depending on usage circumstances [25]. Information on the amount of the fee for the online intervention is kept secret for competitive reasons and therefore not available for the German health care market.

All assessed data were based on participants’ retrospective self-reports, collected via an online survey platform at baseline, after three months (post-assessment) and after six months (follow-up assessment). The recall periods ranged from six months at baseline to three months during the post- and the follow-up assessment.

For data collection on healthcare utilization, we used a modified version of the FIMA [26], a standardized questionnaire which originally was designed for the assessment of health-related resource use within the older population groups in cross sectional and longitudinal surveys. For the purpose of this study, we adapted the recall-periods of FIMA and extended the list of assessed medical services by specific psychiatric and psychotherapeutic treatments. The complete list of assessed medical services is presented in Table 1.

**Table 1:** Assessed medical services by health care sector and corresponding valuation rates adapted from Bock et al. [28], indexed for 2014^a^

Health care sector	Unit of measure	Service/service provider	Valuation rate
Outpatient medical care	Number of contacts	General practitioner	€20.20
		Psychiatrist/psychologist	€45.03
		Psychotherapist/psychotherapy	€78.63
		Neurologist	€45.03
		Internist	€65.90
		Urologist	€24.87
		Gynaecologist	€30.34
		Surgeon	€43.69
		Orthopaedist	€25.60
		Dermatologist	€19.02
		Ophtalmologist	€35.02
		Dentist	€56.26
Outpatient paramedical services	Number of contacts	Physiotherapy	€16.53
		Logopaedics	€38.86
		Medical pedicure	€27.70
		Homeopathic practitioner/osteopath	€20.12
Inpatient hospital services	Number of days	Inpatient hospital treatment	€579.93
		Inpatient hospital treatment - intensive care unit	€1347.08
		Psychiatric inpatient treatment	€342.09
Rehabilitation	Number of days	Inpatient rehabilitation	€122.70
		Outpatient rehabilitation	€47.01
Sickness benefit	Number of days	Incapacity to work	€45.01^ab^

^a^Bock et al. (2015) do not present any unit cost prices for calculating the amount of sickness benefit. The valuation for sickness benefit derives from a large routine-data analysis of more than 3.000 patients with depressive disorders of a major German sickness fund, conducted by the University of Bielefeld (paper under revision)

^b^long-term disability more than 60 days

In order to monetarily value the assessed resource use, the quantitative data on utilization of services were priced, using standardized unit costs from Bock et al. [27]. Costs were calculated by multiplying the units of resource utilization with corresponding unit cost prices and are expressed in euro. For further analyses the single health care costs were summarized to sector related health care costs as presented in Table 1.

Following international standards of health economic evaluation [28], the unit costs from Bock et al. (calculated for the year 2011) were adjusted for inflation. Therefore, all cost-rates were adjusted to inflation for the reference year 2014, based on the German consumer price sub-index for health care [29].

To calculate the medication costs, we used a large database (“Stammdatei^Plus”^) with information on all pharmaceuticals listed in Germany, corresponding active ingredient groups, defined daily doses, pharmacies’ retail prices and more. Since the primary data on medication use were collected between 2012 and 2014, we used the database version 46 with the latest update in December 2014 [30] for our analyses.

On basis of the Stammdatei^Plus^, we calculated drug-specific unit costs per pill, injection, suppository etc. first. Therefore, the pharmacy retail price was used. In a second step, the number of drug units per recall period was calculated for each participant, using the assessed self-reports on dose rate and period of application. Finally, the calculated units of drug use per recall period were combined with the drug-specific unit costs to estimate medication costs.

### Statistical analysis

Before starting with the data analysis, the whole dataset was checked for validity. Whenever reported numbers of resource use (e.g. number of contacts, number of therapy sessions, number of nights spent in hospital) exceeded the maximum number of days of the corresponding recall period, these single implausible data were deleted from the dataset and coded as missing.

The basic data analysis focused on descriptive parameters. We used measures of central tendency and measures of variability to describe differences concerning the sociodemographic variables or healthcare utilization between study groups. To determine the precision of mean values, 95%-confidence intervals were calculated. Additionally, chi-square tests were utilized for further examination of observed group differences.

To describe the assessed outcomes in variation of time and to check the observed values for regularities, time series analyses were conducted. In cases of normally distributed data, a paired t-test was carried out. If this condition was not fulfilled, a Wilcoxon signed-rank test was used. Comparative subgroup-analysis (especially between IG and CAU) were applied using t-test for independent samples. In order to check whether the intervention also has an influence on the costs independently from baseline costs, we conducted a difference in differences analysis. Therefore, the difference in costs between baseline and the study period was calculated. The changes in mean costs were then examined for differences between study groups, using paired t-tests for independent samples. The corresponding h_0_-hypothesis to be tested was: there are no significant differences in changes of mean costs between interventions and controls. Our statistical analyses are based on all available data (pairwise deletion), as this method has the advantage of using all observed data of each subject and leads to unbiased estimations. We did therefore not impute missing values as the used statistical methods are robust and valid for missing at random data. Besides, complete case analysis is the most common way of handling missing data in the analysis of clinical RCTs [31].

To enable a direct comparison of health care expenditures and sickness benefits in variation of time, all costs were calculated for a six-month period. Therefore, the health-care expenditures during the post-assessment period were summed up with those at follow-up assessment. Thus, the presented cost-analyses refer to the time-periods “six months pre enrollment” (baseline) versus “six months post enrolment” (post-assessment and follow-up assessment).

All statistical analyses were performed using IBM SPSS Statistics for windows version 22.0 and R version 3.3.2. The calculation of medication costs was conducted with Microsoft Excel 2016. The final cost variables were reimported to IBM SPSS-statistics for further analyses.

## Results

### Participant flow

**Fig. 1:**
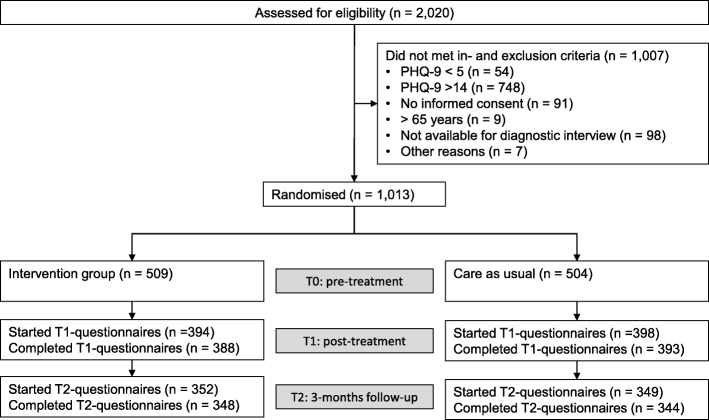
CONSORT participant flow chart

Participants were enrolled in the study between August 2012 and December 2013. Of the 2020 screened subjects, 1007 (49.9%) did not meet the inclusion criteria. Most of them were excluded because they reached a score on PHQ-9 of 14 points or more (*n* = 748; 74.3%). Finally, 1013 participants were randomized into the study groups: 509 to intervention and 504 to care as usual group. The post-assessment-questionnaire was completed by 781 participants (77.1%), 692 (68.3%) completed the 3-months follow-up questionnaire. There were no significant differences in rates of attrition at post treatment or 3-months follow-up between groups. Further, a logistic regression analysis concluded, that neither randomization group nor age, sex, family status, educational status, baseline PHQ-score, baseline diagnosis of depression or panic disorder were significantly associated with dropout status [22]. To obtain full information on participant flow, see the CONSORT flow chart (Fig. 1).

### Participant characteristics

Detailed descriptive statistics for socio-demographic characteristics are presented in Table 2.

**Table 2:** Sociodemographic characteristics at baseline

	Intervention (*n* = 509)		CAU (*n* = 504)		Total (*n* = 1.013)	
	n	%	n	%	n	%
Gender						
Male	159	31.2%	159	31.5%	318	31.4%
Female	350	68.8%	345	68.5%	695	68.6%
Age						
Mean (SD)	42 (11.1),		42 (10.9)		42 (11.0)	
Marital status						
Married	203	39.9%	222	44.0%	425	42.0%
Married, but living separated	12	2.4%	16	3.2%	28	2.8%
Single	118	23.2%	129	25.6%	247	24.4%
In relationship	106	20.8%	83	16.5%	189	18.7%
Divorced	65	12.8%	50	9.9%	115	11.4%
Widowed	5	1.0%	4	0.8%	9	0.9%
Highest academic qualification^a^						
Not yet graduated	2	0.4%	0	0.0%	2	0.2%
No graduation	1	0.2%	0	0.0%	1	0.1%
Lower secondary	29	5.7%	24	4.8%	53	5.2%
Middle secondary	131	25.7%	112	22.2%	243	24.0%
Higher secondary	87	17.1%	85	16.9%	172	17.0%
Highest secondary	249	48.9%	271	53.8%	520	51.3%
Other	10	2.0%	12	2.4%	22	2.2%
Employment status^b^						
Full time	220	44.4%	214	43.5%	434	44.0%
Regular part-time	117	23.6%	114	23.2%	231	23.4%
Mini-Job	0	0.0%	2	0.4%	2	0.2%
Temporary employed	20	4.0%	25	5.1%	45	4.6%
Retraining	7	1.4%	2	0.4%	9	0.9%
Maternity leave/ parental leave/ other absence	14	2.8%	7	1.4%	21	2.1%
Not working	117	23.6%	128	26.0%	245	24.8%

^a^Highest academic qualification according to the German classification: “Hauptschule” (“lower”, 9 years, until age 15/16), “Realschule” (“middle”, 10 years, until age 16/17), “Fachhochschulreife” (“higher”, 12 years, until age 17/18), “Abitur” (“highest”, 12 or 13 years, until age 17–19)

^b^Multiple answers possible

About two-thirds (68.6%) of the 1013 participants who completed baseline questionnaire were female. The mean age of the sample was 44 years at baseline, ranging from 18 to 65 years.

Nearly 60% reported to be married or to have a steady relationship. Approximately the same number of participants stated to work on a regular contract (44.0% full-time, 23.4% part-time). No differences between study groups were found for any of the sociodemographic variables (gender: χ^**2**^ = 0.011, *p* = 0.915; age: t_df = 1011_ = 0.15, *p* = 0.883; material status: χ^**2**^ = 6.753; *p* = 0.240; highest academic qualification: χ^**2**^ = 5.647, *p* = 0.447, employment status: χ^**2**^ = 2.793, *p* = 0.940). This indicates that randomization had been successful.

### Health-related resource use

At baseline-assessment, there was no significant difference in health-related resource use between participants of the intervention group and those receiving care as usual, indicating that randomization was well balanced for resource use as well. Listed medical services or treatments. Briefly, about 80% of the participants in both groups reported that they took at least one medication during the past six months, 85% consulted a general practitioner, about 35% received psychotherapy and nearly 6% had an inpatient hospital stay.

Table 3 presents the percentage of subjects who reported to have used the listed medical services or treatments. Briefly, about 80% of the participants in both groups reported that they took at least one medication during the past six months, 85% consulted a general practitioner, about 35% received psychotherapy and nearly 6% had an inpatient hospital stay.

**Table 3:** Resource use by health care sector and study condition, six months pre-enrollment versus six months post-enrolment

		6 months pre-enrollment						6 months post-enrollment					
		Intervention (*n* = 509)		CAU (*n* = 504)		Between-group differences		Intervention		CAU		Between-group differences	
		%	n	%	n	χ^2^	*p*	%	n (n_total_)	%	n (n_total_)	χ^2^	*p*
	Medication	80.7	411	81.3	410	0.060	0.807	81.4	285 (350)	78.1	271 (347)	1.198	0.274
Outpatient medical care	General practitioner	85.9	437	85.9	433	0.001	0.979	79.3	279 (352)	82.8	289 (349)	1.434	0.231
	Psychiatrist/psychologist	30.1	153	31.5	159	0.263	0.608	34.7	122 (352)	32.7	114 (349)	0.312	0.576
	Psychotherapist	36.1	184	33.5	169	0.764	0.382	38.9	137 (352)	42.4	148 (349)	0.883	0.347
	Neurologist	14.3	73	17.1	86	1.418	0.234	16.2	57 (352)	18.6	65 (349)	0.721	0.396
	Psychiatric day care unit	3.7	19	4.8	24	0.660	0.417	4.3	15 (352)	4.6	16 (349)	0.430	0.835
	Internist	17.1	87	15.7	79	0.371	0.542	21.9	77 (352)	16.0	56 (349)	3.873	0.490
	Gynecologist/urologist	40.5	206	39.3	198	0.149	0.700	33.8	119 (352)	37.2	130 (349)	0.907	0.341
	Surgeon and/or orthopedist	18.9	96	20.6	104	0.503	0.478	23.3	82 (352)	16.6	58 (349)	4.888	0.027
	Dermatologist	19.3	98	17.7	89	0.428	0.513	19.3	68 (352)	17.8	62 (349)	0.280	0.597
	Ophthalmologist	14.7	75	16.1	81	0.347	0.556	19.3	68 (352)	16.6	58 (349)	0.866	0.352
	Dentist	62.1	316	59.5	300	0.696	0.404	54.5	192 (352)	59.3	207 (349)	1.624	0.203
	Outpatient hospital treatment	12.6	64	12.9	65	0.240	0.877	8.5	30 (352)	11.7	41 (349)	2.003	0.157
Outpatient paramedical services	Physiotherapy	31.2	159	29.6	149	0.335	0.562	36.5	126 (345)	27.8	95 (342)	6.018	0.014
	Logopedics	0.8	4	0.6	3		1.000^a^	0.9	3 (345)	0.9	3 (342)		1.000^a^
	Medical pedicure	5.5	28	5.8	29	0.310	0.861	6.1	21 (345)	7.6	26 (342)	0.619	0.431
	Homeopathic practitioner/osteopath	11.8	60	11.3	57	0.570	0.812	14.5	50 (345)	12.9	44 (342)	0.385	0.535
Inpatient hospital treatment	Inpatient hospital treatment	7.3	37	10.7	54	3.676	0.055	6.7	23 (345)	6.7	23 (342)	0.001	0.976
	Psychiatric inpatient treatment	6.9	35	7.7	39	0.278	0.598	2.0	7 (345)	4.7	16 (342)	3.726	0.540
Rehab	Outpatient	0.6	3	1.6	8	2.463	0.292	1.4	5 (345)	1.2	4 (342)	2.154	0.341
	Inpatient	6.3	32	6.7	34			5.8	20 (345)	3.5	12 (342)		
	Sickness benefit	9.5	48	12.2	61	1.947	0.163	7.0	24 (352)	9.5	32 (349)	1.324	0.250

^a^Fisher’s exact test

Compared to baseline, the mean percentage of participants reporting to have received the assessed medical services six months post enrollment did not differ significantly, neither in variation of time nor between study groups. An exception is treatment by a surgeon or an orthopedist. Whereas the percentage of participants who consulted a surgeon or an orthopedist increased by 4.4%age points from 18.9% at baseline to 23.3% six months post enrollment in the intervention group, it decreased by 4.0 percentage points within the care as usual group (20.6% to 16.6%; between-group difference post-enrollment: χ^**2**^ = 0.011, *p* = 0.915).

### Incapacity for work

**Fig. 2:**
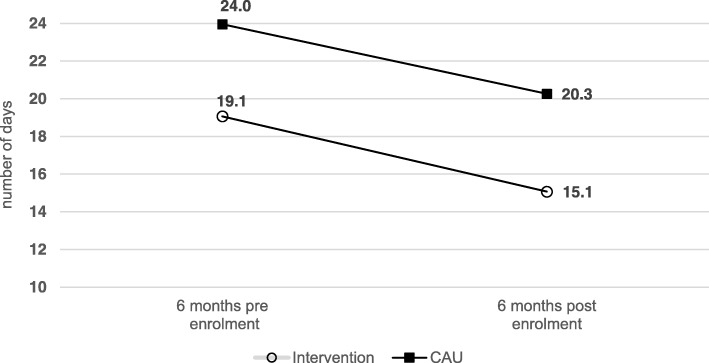
Duration of incapacity for work six months pre enrollment compared to six months post enrollment

In both study groups, the duration of incapacity for work decreased significantly during the period of six months post enrollment compared to six months pre enrollment (see Fig. 2). The average number of days of incapacity to work decreased by 4 days in both groups, whereas participants of the control-group reported a significant higher mean duration of incapacity for work during the six weeks pre enrollment to the study as well as during the six months post enrollment (IG_pre-post_: 19.1 to 15.1 days, − 20.9%, *p* = 0.001; CAU_pre-post_: 24.0 to 20.3 days, − 15.4%; *p* = 0.026). The changes in duration of incapacity for work did not differ significantly between groups (t_df = 640_ = 0.74; *p* = 0.462).

### Costs

**Fig. 3:**
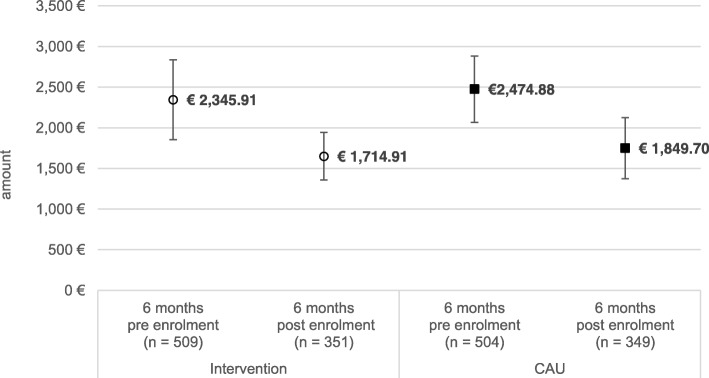
Total health care expenditure six months pre versus six months post enrollment

There were no significant differences in total direct health care costs between the study conditions at baseline (see Table 4). Six months pre enrolment mean total direct costs per participant were €2346 in the intervention group and €2475 in the care as usual group, showing only a small difference of €129 (t_df = 980_ = 0.35; *p* = 0.730). During the six months after enrollment in the trial, mean total costs decreased significantly in both study groups. In the intervention group, total costs decreased by €631 (t_df = 412_ = 1.70; *p* = 0.002), in the care as usual group average costs decreased by €625 (t_df = 420_ = 4.004; *p* = 0.000) (see Fig. 3). The average reduction in total costs did not significantly differ between study conditions (t_df = 579_ = − 1.77; *p* = 0.139).

**Table 4:** Health care expenditures (in €) by sector and study condition, six months pre-enrollment versus six months post-enrollment

	Intervention						CAU						*p*-value between-group differences^#^
	Mean	5% trimmed mean	95% - CI of the mean			*p*-value within-group differences^#^	Mean	5% trimmed mean	95% - CI of the mean			*p*-value within-group differences	
Total amount													
6 months pre enrollment	2345.91	1506.42	1842.50	–	2784.98	0.002*	2474.88	1705.61	2032.64	–	2820.61	0.000*	0.730
6 months post enrolment	1714.91	1681.41	1772.44	–	2525.51		1849.70	1021.75	1231.78	–	1973.36		0.139
Medication costs													
6 months pre enrollment	277.50	33.17	34.25	–	589.26	0.007*	276,96.	45.83	90.82	–	463.09	0.634	0.998
6 months post enrolment	108.41	26.09	20.09	–	196.74		154.91	19.45	−43.55	–	353.36		
Outpatient medical care													
6 months pre enrollment	749.70	585.73	647.40	–	852.0	0.144	683.85	581.25	611.54	–	756.16	0.099	0.302
*… thereof psychotherapy*	*477.32*	*313.54*	*382.85*	*–*	*571.8*	*0.031*	*405.93*	*304.76*	*342.60*	*–*	*469.25*	*0.164*	*0.218*
6 months post enrolment	716.69	634.68	637.16	–	796.2		762.41	670.20	673.70	–	851.12		0.036*
*… thereof psychotherapy*	*397.24*	*325.58*	*333.67*	*–*	*460.8*		*465.78*	*370.32*	*387.18*	*–*	*544.39*		0.008*
Outpatient paramedical services													
6 months pre enrollment	76.24	53.42	62.10	–	90.37	0.036*	85.66	58.31	69.89	–	101.43	0.411	0.382
6 months post enrolment	99.90	75.82	81.99	–	117.81		79.99	48.14	59.05	–	100.94		0.031*
Inpatient hospital treatment													
6 months pre enrollment	646.97	61.35	374.47	–	919.48	0.484	680.32	135.49	421.59	–	939.04	0.075	0.862
6 months post enrolment	374.25	15.63	170.19	–	578.32		468.91	30.01	207.61	–	730.21		0.427
Rehabilitation													
6 months pre enrollment	285.82	39.4	181.9	–	389.8	0.820	297.20	65.61	199.61	–	394.79	0.161	0.875
6 months post enrolment	275.96	36.3	145.8	–	406.1		188.64	0.00	84.10	–	293.18		0.484
Sickness benefit													
6 months pre enrollment	309.68	78.27	212.65	–	406.70	0.005*	450.89	197.72	328.94	–	572.85	0.000*	0.075
6 months post enrolment	139.70	24.89	81.30	–	198.10		194.84	65.89	126.21	–	263.47		0.111

#t-test for independent samples; pre enrollment: comparison of mean values, post enrollment: comparison of differences in mean costs between pre and post enrollment

*p ≤ 0.05

Besides, on closer examination of sector-specific health care costs we found some important differences between the study groups: Whereas the mean direct costs of outpatient treatment slightly decreased in the intervention group, the average per participant costs increased in the care as usual group (see Table 4). Thus, average outpatient health care costs decreased by 4.4% (€33) from €750 to €717 in the intervention group (t_df = 344_ = 1.47; *p* = 0.144), while costs increased about 11.5% (€78) in the control group from €684 during the period of six months before enrollment to €762 six months after enrollment to the trial (t_df = 340_ = − 1.65; *p* = 0.099). These changes in average outpatient costs did significantly differ between groups (t_df = 684_ = 2.16; *p* = 0.036). The increase in outpatient health care costs in the control group could mainly be traced back to a rise in costs of psychotherapeutic treatment: the six months pre enrollment costs for utilization of psychotherapy increased by €60 on average during the six months post enrollment. By contrast, mean costs for psychotherapeutic treatment decreased by €80 in the intervention group. The described contrary trend in costs for utilization of psychotherapeutic treatment lead to a significant difference in the development of mean changes between intervention group and care as usual (t_df = 685_ = 2.57; *p* = 0.008).

In comparison to the described development of outpatient health care cost, an opposing trend could be identified when analyzing changes in cost for outpatient paramedical services. Whereas the mean direct costs for outpatient paramedical services slightly decreased in the care as usual group by 6.6% (€6) on average, costs for utilization of outpatient paramedical services increased in the intervention group by 31.0% (€24). Even if the change in costs between baseline and post enrollment was significant only for the intervention group (see Table 4), the difference in development of costs between the study groups was statistically significant (t_df = 684_ = 2.16; *p* = 0.031). The significant increase in costs for outpatient paramedical services in the intervention group was mainly caused by an increase in utilization of physiotherapeutic treatment. The average cost per participant decreased about 43.5% (€27), from €60 six months pre enrollment to €87 at six months post enrollment.

Costs for inpatient hospital treatment as well as rehabilitation costs decreased between baseline and six months post enrollment in both study groups. There were no significant differences between study conditions, neither at baseline or post-enrollment nor in mean changes of costs.

Medication costs also decreased in both groups, but the reduction in average costs did not significantly differ between interventions and controls (t_df = 789_ = 1.74; *p* = 0.998).

## Discussion

### Principal findings

In this study we have not found that the internet intervention has a significant effect on the percentage of people reporting to have received single healthcare services, on incapacity for work due to illness and resulting *total* direct costs from payer perspective.

Therefore, the partially perceived changes in sector-specific health care costs can be traced back to changes in rates of utilization: decreasing costs as observed in the field of inpatient treatment, rehabilitation or drug expenses, result from shortened durations of stay, reductions in the number of perceived drugs, variations in dosage or shortened periods of medication use.

Numeric differences in health care expenditures between study groups existed in outpatient treatment costs. Whereas the mean direct costs of outpatient treatment slightly decreased in the intervention group (4.4%; €33), the average per participant costs increased in the care as usual group (11.5%; €78). The significant difference in change of outpatient treatment costs could be attributed to differences in changes of costs for psychotherapeutic treatment from baseline. Whereas costs for psychotherapeutic treatment decreased in the intervention group by 16.8% (€80) during the period of six months post enrollment to the study, costs increased in the control group by 14.7% (€60) (see Table 4).

The higher average duration of incapacity for work which was observed in the intervention group pre and post enrollment to study cannot be explained by baseline differences in sociodemographic characteristics, in severity of depressive symptoms or in the health-related resource use. As part of the RCT-design, participants were randomly assigned to the two study groups. It should therefore be presumed that the observed higher level of incapacity for work in the intervention group has a random origin. The simultaneous reduction in duration of incapacity for work in both study groups does not allow any indication that the use of internet intervention has led to a significant reduction in absenteeism.

### Previous studies

Our estimated total direct health care costs are higher than average direct health care costs of other previously performed economic evaluations. To the best of our knowledge, for the German context only two studies exist, which give a detailed description for total direct per patient costs of depression. Whereas the estimated annual direct treatment costs in these studies differ between €686 [[32] and €3849 [33] per patient, the estimated total cost for a period of only six months differs between about €2350 to €2500 at baseline and €1700^1^ to €1850 six months post assessment within our study (see Table 1). It has to be noted, that the methodology of cost assessment and cost calculation in both studies differed from our approach. The first study focused on average direct health care costs of non-institutionalized adults with depression. In contrast to our study, rehabilitation costs, outpatient paramedical services and sickness benefit were not taken into account, which could be an explanation for the significantly lower estimated average costs. Furthermore, because of the restriction to the non-institutionalized population costs might be underestimated [32]. The second study assessed service utilization (including inpatient, outpatient and rehabilitative services) and total direct costs of care in patients with depressive disorder. Again, outpatient paramedical services and sickness benefit were not included in the cost calculation. Additionally, the assessment of medication costs was limited to the depression-specific treatment, prescriptions due to somatic illness were not taken into account [33].

A further difference between our study and previously published studies regards to the instruments used to assess the health-related resource utilization. A recently published meta-analysis on cost-effectiveness of internet-based interventions for the treatment of depression [18] provides an overview of the cost assessment instruments that were used within twelve trials focusing on the cost-effectiveness of e-mental-health interventions. Half of the studies used the “Trimbos and Institute of Medical Technology Assessment Cost Questionnaire for Psychiatry” (TiC-P) to assess direct medical and non-medical costs [18]. The TiC-P allows for the measurement of medical costs as well as productivity losses in patients with a mental disorder. In contrast to the FIMA the TiC-P only focusses on the assessment of contacts within the *mental* healthcare sector and the use of medication [34]. The FIMA-questionnaire, on the other hand, covers total costs of care.

Several studies have documented that (acute) psychiatric disorders are associated with an increased use of primary care resources but also with a more frequent use of outpatient specialist care [9, 35]. Against this background, the FIMA-questionnaire provides more comprehensive information on healthcare utilization. This could be another reason for the higher estimated costs values within our study.

In conjunction with further differences in cost categorization approaches or differences within the study sample the illustrated differences in survey methods and used instruments limit the comparability of this trial with previously published studies.

A strength of our study is the large sample size. In comparison to other published studies in this research area our study benefits from the huge number of participants enrolled in the trial. To the best of our knowledge the present health economic evaluation is the first published one, which was conducted alongside a prospective randomized controlled trial with a sample size of more than 1000 participants [18]. Another advantage is the wide range of recruitment settings, varying from outpatient to inpatient settings as well as from specialized web-based communication platforms for people with depressive disorders to media used by the general population. The wide range of recruitments settings contributes the extern validity of the present research and supports the hypothesis, that the intervention is effective across different recruitment sources including clinical settings, which could be demonstrated by Klein et al. in another analysis in the context of the EVIDENT-trial [36].

One aspect less focused within this manuscript is the clinical efficacy of deprexis. However, since the medical benefits are of great importance in the context of health economic evaluations, it should be noted that different studies along randomized controlled trials as well as a meta-analysis provided evidence for the clinical effectiveness of deprexis. Thus, a comparison from eight studies demonstrated the effectiveness of deprexis for depressive symptoms at post-intervention, with a medium effect size of g = 0.54 (95% CI: 0.39–0.69) [25]. The results of the EVIDENT-trial also confirm the clinical effectiveness of the program. The study was able to demonstrate that internet intervention was superior to CAU alone in reducing mild to moderate depressive symptoms [22].

### Limitations

Some limitations should be considered when interpreting the results of our study. First, our health economic evaluation was conducted alongside a large multicenter study which was primarily designed to test clinical effectiveness of the internet intervention deprexis. Therefore, the EVIDENT-trial was powered to detect a post-treatment group difference in depression on the main outcome variable PHQ-9 [23]. Possible savings due the use of deprexis were not taken in account when calculating the sample-size. This could be a potential reason for the rather small and mostly not significant differences in direct costs between study groups. Furthermore, the relatively short follow-up period of six months could be another reason for only little differences in health care utilization, medication use, absenteeism due to sickness and resulting costs. Thus, further research is needed to assess the long-term health economic effects (at least two or three years of follow up) of e-mental-health interventions like deprexis.

All collected data were based on patients’ self-reports. Even though we used a standardized instrument for the evaluation of participants’ resource utilization, the assessed health related resource use may suffer from under-reporting due to memory failure or over-reporting due to recall bias. For the purpose of this study, the recall time frames of the FIMA (seven days for medication use, three months for outpatient medical care and outpatient paramedical services and 12 months for inpatient hospital treatment and rehabilitation) had to be standardized and adjusted to the general follow-up dates of the multicentre trial (six months at baseline and three months at post-treatment and follow-up). The relatively long recall periods, especially the period of six months at baseline, may strengthen these effects. As recent studies revealed, the reliability of collected data is clearly restricted by the degree to which patients accurately recall quantities of resources used [34, 37, 38]. Hence, different methods have been suggested to prevent reporting-bias or to correct assessed primary data for recall bias. The most common ways suggested for solving these problems are using patient controls as well as blinding the participants for the study hypothesis being tested [39], which both was realised in the present study.

Another limitation exists with regard to the transferability of the study results. Firstly, study enrollment was limited to people aged between 18 and 65 years as well as to those with mild to moderate depressive symptoms. Therefore, the results cannot be generalized to older people or patients with severe depression. In comparison to the corresponding German general population, participants in the EVIDENT-trial had a higher educational level and women were overrepresented in the study (68.6% females vs. 31.4 percent males) [40–42]. As Späth et al. demonstrated, these findings are in line with previous studies [42]. Thus, the higher proportion of women can be explained by a twice as high prevalence of moderate depression among women compared to men. Furthermore, women seem to be more likely to seek help then men, as a cross-sectional study on patterns of lay and professional help-seeking in men and women showed [42, 43]. The higher educational level within this trial could be explained by a higher demand for internet interventions in people with a higher educational level, which was shown for users of a web-based computer-tailored intervention promoting heart-healthy behaviours [44].

One further limitation is that the interventions costs could not be included to the analysis, as this economic evaluation was derived from payer perspective and negotiated license-fees for health insurance companies are not published. The only publicly available price-information relates to a single license for private persons (use of deprexis over a period of 90 days) which amounts to €297.50 including value-added tax [45]. Providing framework contracts with health insurance companies, the program-fees from payer perspective can be assumed to be significantly lower than those for individuals. As shown in Table 4, there is a statistically not significant difference of €135 in mean total costs at six months post-enrollment. It is possible that this difference in total costs could be offset if the program costs are taken into account.

A last limitation that should be noticed is that the conducted costing methodology is only able to generate approximatively information on direct health care costs. The utilization of unit costs for the monetarily valuation of health-related resource use cannot express actual per participant costs. To give an example: costs for inpatient hospital treatment were calculated by multiplying the number of days spent at hospital with a unit cost rate per day, whereas reimbursement within the German inpatient hospital sector is based on fees per case. Furthermore, the reimbursement-system in the outpatient sector combines elements of capitation payment with those of fee for service payments. However, costing by unit costs is a common and approved methodological approach for calculating health care costs within health economic evaluation studies [37, 46].

## Conclusion

The present study indicates the health economic potential of innovative e-mental-health programs. Our results suggest that the simultaneous use of web-based self-help programs for depression in combination with care as usual leads to a significant decrease in outpatient treatment costs, especially in those related to different types of psychotherapeutic treatment. With regard to the strong evidence for the clinical effectiveness of deprexis [22], we would recommend the use of this program when weighing up cost and benefits. Although no significant savings in total health care costs could be demonstrated, from a health economic perspective the gained clinical benefits are a strong argument for the use of the cognitive behavioral self-help program.

Considering the above-mentioned limitations of our study, further research on health economic effects of innovative internet programs in the treatment of depressive disorders seems necessary to verify our findings by addressing remaining methodical limitations as well as to widen evidence for (positive) health economic effects such as savings in direct and indirect health care cost.

## Data Availability

The data that support the findings are not publicly available, as the publication of the collected primary data is not covered by the informed consent.

## Abbreviations

EVIDENT-Trial: EffectiVeness of Internet-based DEpressioN Treatment
FIMA: Questionnaire for Health-Related Resource Use in an Elderly Population
PHQ: Patient Health Questionnaire
RCT: randomized controlled trial
TiC-P: Trimbos and Institute of Medical Technology Assessment Cost Questionnaire for Psychiatry

## Footnotes

1 Total amount of direct health care costs in the intervention group without costs of the intervention (value therefore differs from that shown in Table 1).
